# Association between left atrial reverse remodeling and maintenance of sinus rhythm after catheter ablation of persistent atrial fibrillation

**DOI:** 10.1007/s00380-019-01475-1

**Published:** 2019-07-25

**Authors:** Yoshihiko Kagawa, Eitaro Fujii, Satoshi Fujita, Masaaki Ito

**Affiliations:** Department of Cardiology and Nephrology, Mie University Graduate School of Medicine, 2-174 Edobashi, Tsu, 514-8507 Mie Japan

**Keywords:** Persistent atrial fibrillation, Ablation, Left atrial reverse remodeling, Prognostic factor

## Abstract

The success rate of catheter ablation of persistent atrial fibrillation (AF) is not satisfactory, for reasons that are unclear. The purpose of this study was to examine the relationship between left atrial reverse remodeling after ablation and recurrence of AF in patients with persistent AF. One hundred and thirty-two patients with persistent AF were enrolled. Extensive encircling pulmonary vein isolation plus ablation of complex fractionated atrial electrograms was performed. Bepridil or amiodarone was prescribed for 3 months after ablation. All patients were studied by serial echocardiography and 24-h ambulatory electrocardiogram at baseline, for the day after ablation, and at 1-, 3-, and 6-month intervals after ablation. Recurrence of AF was observed in 42 patients at 2-year follow-up. The duration of AF (median 12 (IQR 6–37) vs 8 (IQR 5–17) months, *p* < 0.05), and early recurrence of AF (69 vs 26%, *p* < 0.05) after ablation were significantly different between the patients with AF recurrence and those without. The left atrial dimensions at 3 months (40 ± 6 vs 44 ± 6 mm, *p* < 0.001) and 6 months (40 ± 6 vs 44 ± 6 mm, *p* < 0.001) were significantly smaller than those just after ablation in the patients without AF recurrence. A 5% reduction from baseline in the left atrial dimension at 6 months after ablation was associated with freedom from late AF recurrence (*p* < 0.05). Left atrial reverse remodeling after ablation of persistent AF was associated with freedom from late recurrence of AF.

## Introduction

Pulmonary vein isolation is an established and effective treatment for patients with paroxysmal atrial fibrillation (AF) [1]. However, the success rates of catheter ablation for patients with persistent AF are not satisfactory, for reasons that are unclear. Intensive ablation of the left atrium (LA) including complex fractionated atrial electrogram (CFAE) ablation combined with extensive pulmonary vein isolation (EPVI) improves outcomes of catheter ablation in patients with persistent AF [2], but a substantial portion of these patients experience relapse of AF. The STAR AF II trial clearly showed that additional substrate modification (CFAE or linear lesions) following EPVI offers no benefit in AF reduction [3]. A critical issue is how to identify patients in whom sinus rhythm maintenance can be achieved after catheter ablation. In some cases, the size of LA decreases after catheter ablation, which is considered to indicate improvement of LA remodeling [4]. We assessed the hypothesis that atrial reverse remodeling after catheter ablation would be associated with maintenance of the sinus rhythm in patients with persistent AF.

## Methods

### Study population

This case–control study included 132 consecutive patients with persistent AF. All patients underwent AF ablation between April 2012 and April 2016, and were followed up for 2 years. AF was classified as persistent if there was a failure to self-terminate within 7 days. Long-standing persistent AF was defined as AF lasting for more than 1 year. This study was approved by the Mie University Hospital Institutional Review Boards (reference number: 3038), and all patients gave their written or opt-out informed consent.

### Ablation procedure

After informed written consent was obtained, an electrophysiological study was performed in the postabsorptive state under light sedation [5]. Trans-esophageal echocardiography was performed to exclude the possibility of LA thrombus just before ablation in all patients [6]. After internal jugular and femoral vein punctures, a heparin bolus (100 U/kg) was administered, and continuous infusion of heparin was provided thereafter to maintain an activated clotting time of between 250 and 350 s. A diagnostic duodecapolar catheter was placed in the coronary sinus via the jugular vein. Three long sheaths were inserted through the femoral vein and introduced in the LA through a single transseptal puncture guided by intracardiac echocardiography. Two eicosapolar circumferential catheters (Lasso 2515, Biosense Webster, Diamond Bar, CA, USA and Optima 2015, St. Jude Medical, St. Paul, MN, USA) were introduced in the LA through the transseptal long sheaths, and an ablation catheter with a 3.5-mm irrigated tip (EZ Steer Thermocool, Biosense Webster, Diamond Bar, CA, USA) was introduced in the LA. All imaging was performed using a biplane flat-panel detector angiographic suite (Allura Xper FD10/10 angio system; Philips Healthcare, Best, Netherlands). Intra-procedural three-dimensional (3D) rotational angiography of LA (3D-ATG) was performed with 15 mL of contrast medium + 15 mL of saline injected by hand directly into the bilateral upper pulmonary veins via the Swartz long sheaths placed in the left and right superior pulmonary veins during rapid ventricular pacing at 200 beats per minute. 3D-ATG was performed from the right oblique at 59° and to the left oblique at 100° of C-arm rotation at a sampling rate of 30 frames per second for a total of 3.8 s [7–9]. After the rotational angiography, the data were automatically transferred from the FD10/10 detector System to the EP Navigator Workstation. The 3D LA-PV images were reconstructed using the standard 3D reconstruction algorithms available on the EP Navigator Workstation within 1 min, and were seamlessly integrated into the CARTO^®^ system using the local area network in our hospital, within 2–3 min [9]. The 3D LA-PV image was then integrated with the live fluoroscopy. Extensive encircling pulmonary vein isolation [10] plus ablation of complex fractionated atrial electrograms (CFAE) [2, 11] was performed. Radiofrequency energy was delivered with a target temperature of 40 °C, at a power setting of 25–30 W. Each target site was ablated for 20–30 s. The endpoints of ablation were complete elimination or dissociation of PV potentials determined using the circumferential catheter and no further CFAE observed at the targeted sites.

### Echocardiography

Transthoracic echocardiography was performed at baseline, the day after ablation, and at 1-, 3-, and 6-month intervals after ablation. LA diameter was measured through antero-posterior diameter from the parasternal long-axis view. The LV ejection fraction (LVEF) was assessed by Simpson’s method. The ratio of peak early (*E*) to late (*A*) diastolic transmitral flow velocity (*E*/*A*) was calculated using pulsed Doppler echocardiography. Tissue Doppler imaging (TDI) of the mitral annular motion was obtained from the apical 4-chamber view. The sample volume was placed at the septal and lateral mitral annuli, and early (*e*′) and late (*a*′) diastolic annular velocities were measured. The ratio of E/e′ was calculated. The rate of LA reduction at 6 months after ablation was defined as (LA diameter (post 1 day)-LA diameter (post 6 months))/LA diameter (post 1 day) × 100 (%).

### Follow-up

All patients were monitored in the hospital for at least 2 days after their procedures. Bepridil or amiodarone was prescribed after ablation for 3 months and was then stopped if AF recurrence was not observed. At 1, 3, 6, 12, and 24 months after ablation, patients underwent clinical review and 24-h ambulatory ECG monitoring to identify asymptomatic arrhythmias. Early AF recurrence (ERAF) was defined as any recurrence of AF > 30 s during the first 3 months of follow-up. Late recurrence was defined as any recurrence of AF > 30 s between 3 and 24 months. Oral anticoagulation was discontinued 6 months after ablation in patients with a CHADS_2_ score of ≤ 1.

### Statistical analysis

Continuous variables are expressed as the mean ± standard deviation (SD) or median and interquartile range, and categorical variables as absolute values and percentages. Results were analyzed using the SPSS 22.0 software (SPSS Inc., Chicago, IL, USA). Continuous data were evaluated using the unpaired Student *t* test or nonparametric Mann–Whitney test when dealing with non-normal distributions. Categorical data were analyzed using the Chi squared test or Fisher’s exact test. Kaplan–Meier analysis with the log-rank test was used to determine the probability of freedom from recurrent AF. Multivariate Cox regression analysis was performed to determine the independent predictors of recurrence of AF. A receiver-operating characteristic analysis was performed to define cutoff values, and the cutoff values were defined by minimizing the expression of (1-sensitivity)^2^ + (1-specificity)^2^. Significance was established at *P* < 0.05.

## Results

### Patient characteristics

Patient characteristics are summarized in Table 1. The mean age of the patients was 63 ± 10 years, 107 patients (81%) were male, 70 patients (53%) had hypertension, and 21 patients (16%) had a history of congestive heart failure. The duration of AF was 9 months, and there were 54 patients (41%) with long-lasting AF. The mean CHADS_2_ and CHA_2_DS_2_-VASc scores were 1 ± 1 and 2 ± 2 points, respectively.

**Table 1:** Patient baseline characteristics

Study population (*n* = 132)	Value
Age, years	63 ± 10
Male, *n* (%)	107 (81)
Body mass index, kg/m^2^	23.8 ± 3.7
Duration of AF, months	9 (6–24)
Long-lasting AF, *n* (%)	54 (41)
Hypertension, *n* (%)	70 (53)
Heart failure, *n* (%)	21 (16)
Diabetes mellitus, *n* (%)	19 (14)
Stroke/TIA, *n* (%)	17 (13)
CHADS_2_ score	1 ± 1
CHA_2_DS_2_-VASc score	2 ± 2
BNP, pg/ml	112.9 (65.1–175.0)
eGFR, mL/min/1.73 m^2^	66.7 ± 19.8
ACE-I, *n* (%)	15 (11)
ARB, *n* (%)	44 (33)
Beta-blockers, *n* (%)	56 (42)
Calcium blockers, *n* (%)	48 (36)
Aldosterone blockers, *n* (%)	6 (5)
Alpha-blockers, *n* (%)	1 (1)
Diuretics, *n* (%)	17 (13)
AAD user, *n* (%)	31 (24)

Values are presented as mean ± SD, median (IQR), or *n* (%)

*AADs* antiarrhythmic drugs, *ACE-I* angiotensin-converting enzyme inhibitor, *AF* atrial fibrillation, *ARB* angiotensin receptor blocker, *BNP* B-type natriuretic peptide, *eGFR* estimated glomerular filtration rate, *TIA* transient ischemic attack

### Catheter ablation and clinical outcomes

EPVI was performed in all patients, confirmed by entrance and exit block. Ablation of CFAE was performed in the patients who had persistent AF after PV isolation (*n* = 125, 95%). An additional linear ablation extending from the mitral valve annulus to the junction of the left inferior PV was performed in four cases, and cavotricuspid isthmus ablation was performed on the patients complicated with common atrial flutter (*n* = 10, 8%). Superior vena cava isolation was performed in a few patients with AF recurrence (*n* = 4, 3%). Cardiac tamponade occurred in one patient (0.9%) during PV isolation, necessitating pericardial drainage, and three patients had vasospastic angina just after the ablation procedure [12].

**Fig. 1:**
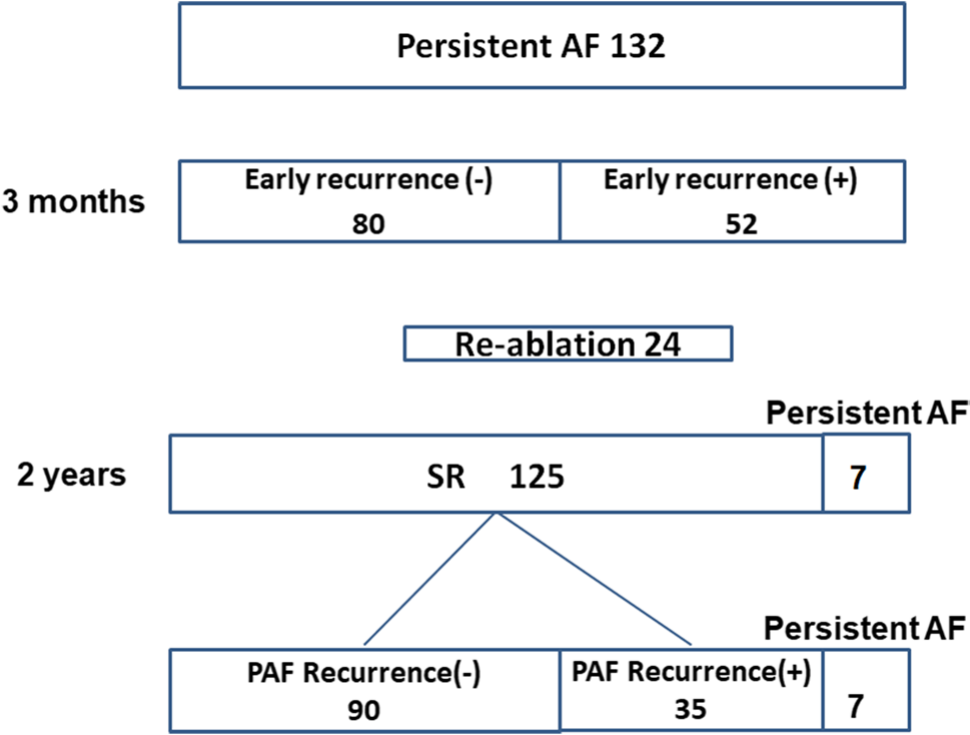
Total results are shown in the flow diagram. *AF* atrial fibrillation, *PAF* paroxysmal AF, *SR* sinus rhythm

During the follow-up period of 24 months, 125 patients (95%) maintained sinus rhythm (SR) and 7 patients (5%) were found to have persistent AF. ERAF occurred in 52 patients (39%) within the 3-month blanking period. Twenty-four patients (18%) underwent re-ablation for AF recurrence. Of the 132 patients, 90 patients (68%) were free from late recurrence of AF and 35 patients (27%) had paroxysmal AF recurrence (Fig. 1).

### Comparison of clinical characteristics between the patients free from AF and those with recurrence

Age, sex, BNP before ablation, a history of hypertension, diabetes mellitus, and strokes were not different between the patients with and without late recurrence of AF (Table 2). Duration of AF (12 (6–37) vs 8 (5–17) months, *p* < 0.05), early recurrence of AF (69 vs 26%, *p* < 0.05), and continuation of antiarrhythmic drugs (57 vs 22%, *p* < 0.05) were significantly different between the patients with and without AF recurrence (Table 2).

**Table 2:** Comparison of clinical characteristics between the patients with and without late recurrence of AF

	Freedom from AF (*n* = 90)	Recurrence of AF (*n* = 42)	*P* value
Age, year	62 ± 10	63 ± 11	0.805
Male, *n* (%)	72 (80)	35 (83)	0.495
Body mass index, kg/m^2^	24.0 ± 3.8	23.3 ± 3.6	0.449
Duration of AF, months	8 (5–17)	12 (6–37)	0.021
Long-lasting AF, *n* (%)	32 (36)	22 (52)	0.073
Hypertension, *n* (%)	49 (54)	21 (50)	0.438
Heart failure, *n* (%)	14 (16)	7 (17)	0.947
Diabetes mellitus, *n* (%)	9 (10)	10 (24)	0.099
Stroke/TIA, *n* (%)	12 (13)	5 (12)	0.773
CHADS_2_ score	1 ± 1	1 ± 1	0.927
CHA_2_DS_2_-VASc score	2 ± 2	2 ± 2	0.773
BNP before ablation, pg/ml	108.7 (64.8–181.5)	124.9 (66.2–173.2)	0.723
eGFR, ml/min/1.73 m^2^	65.0 ± 12.1	69.9 ± 29.7	0.139
ACE-I, *n* (%)	8 (9)	7 (17)	0.251
ARB, *n* (%)	29 (32)	15 (36)	0.784
Beta-blockers, *n* (%)	34 (38)	22 (52)	0.134
Calcium blockers, *n* (%)	35 (39)	13 (31)	0.124
Aldosterone blockers, *n* (%)	1 (1)	4 (7)	0.200
Alpha-blockers, *n* (%)	1 (1)	0 (0)	0.609
Diuretics, *n* (%)	11 (12)	6 (14)	0.884
Continuation of AADs, *n* (%)	20 (22)	24 (57)	0.018
Early recurrence of AF, *n* (%)	23 (26)	29 (69)	0.015

Values are presented as mean ± SD, median (IQR), or *n* (%)

*AADs* antiarrhythmic drugs, *ACE-I* angiotensin-converting enzyme inhibitor, *AF* atrial fibrillation, *ARB* angiotensin receptor blocker, *BNP* B-type natriuretic peptide, *eGFR* estimated glomerular filtration rate, *ERAF* early recurrence of atrial fibrillation, *TIA* transient ischemic attack

### Changes in echocardiographic parameters and BNP

**Fig. 2:**
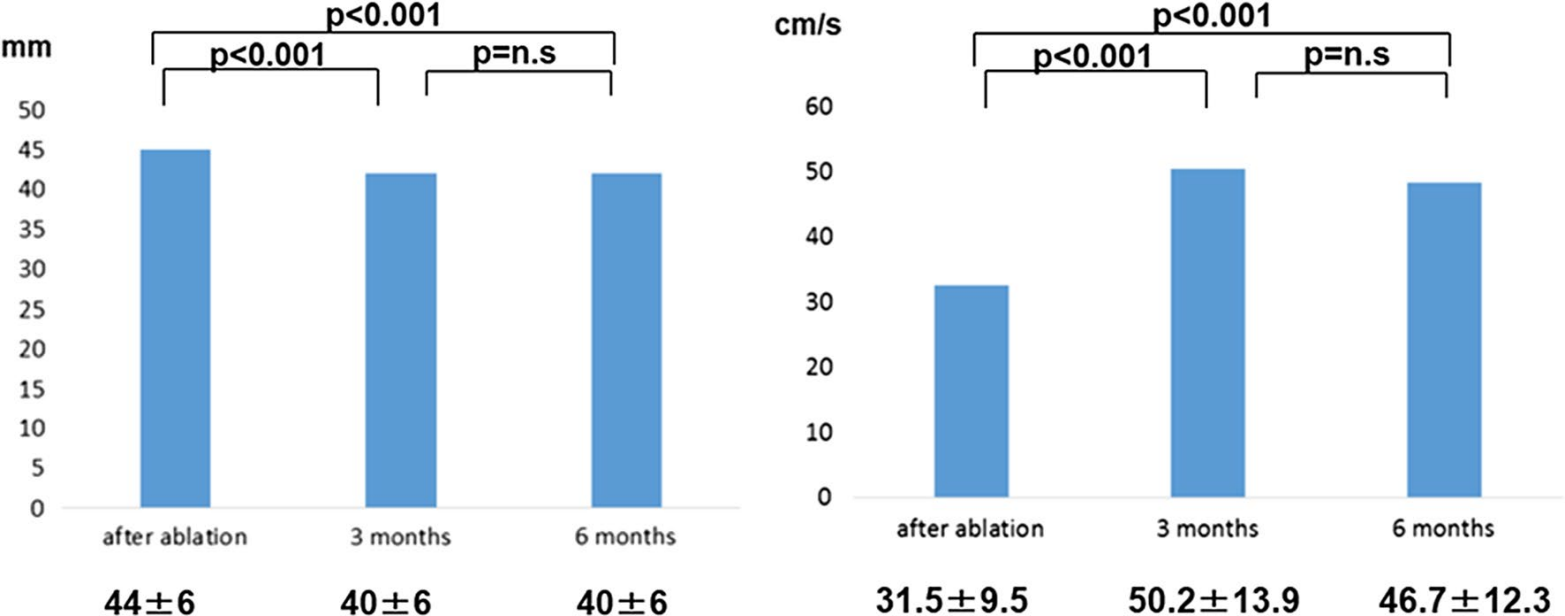
Left panel: LA dimensions at 3 and 6 months decreased after AF ablation in the patients without AF recurrence. Right panel: transmitral A wave values at 3 and 6 months increased after ablation in the patients without AF recurrence. *AF* atrial fibrillation, *LA* left atrium

**Fig. 3:**
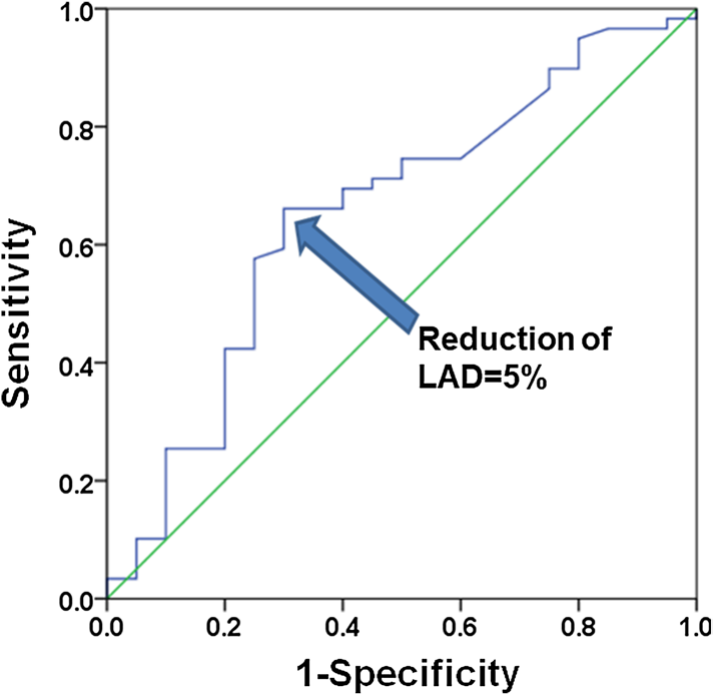
Calculation of an optimal cutoff value using receiver-operating characteristic curve analysis with respect to the reduction rate of the left atrial dimension. The cutoff value of reduction of the left atrial dimension was 5%. The area under the receiver-operating characteristic curve is 0.653 (95% CI 0.51–0.80, *p* < 0.05). *LAD* left atrial dimension

The left atrial dimensions at 3 months (44 ± 6 vs 40 ± 6 mm, *p* < 0.001) and 6 months (44 ± 6 vs 40 ± 6 mm, *p* < 0.001) were significantly smaller compared with just after ablation in the patients without AF recurrence (Fig. 2). The left atrial dimension at 3 months was significantly different between the patients with AF recurrence and without [42 ± 4 vs 40 ± 6 mm (*p* < 0.05)] (Table 3). The A wave amplitudes at 3 and 6 months were greater than just after ablation in the patients without AF recurrence (Fig. 2). There were no differences in the values of LVEF at baseline, at 3, and at 6 months between the patients with and without AF recurrence. There was a significant decrease between the median BNP concentrations at baseline and those at 3 and 6 months (111.0 vs 34.9 pg/ml, *p* < 0.001; 111.0 vs 30.4 pg/ml, *p* < 0.001). The rate of LA reduction at 6 months had a significant relationship with late recurrence (p < 0.05). The cutoff value of the reduction of left atrial dimension was 5% (area under the curve = 0.653, *p* < 0.05) (Fig. 3).

**Table 3:** Echocardiographic parameters

	Freedom from AF (*n* = 90)	Recurrence of AF (*n* = 42)	*P* value
LAD at ablation, mm	44 ± 6	45 ± 6	0.629
LAD at 3 months, mm	40 ± 6	42 ± 4	0.044
LAD at 6 months, mm	40 ± 6	42 ± 5	0.165
A wave at ablation, cm/s	32.8 ± 10.7	32.0 ± 10.6	0.730
A wave at 3 months, cm/s	50.5 ± 14.8	50.3 ± 16.5	0.723
A wave at 6 months, cm/s	48.8 ± 15.8	47.2 ± 20.3	0.614
LVEF before ablation, %	61 ± 11	63 ± 11	0.550
LVEF at ablation, %	64 ± 9	62 ± 11	0.332
LVEF at 3 months, %	66 ± 6	66 ± 9	0.996
LVEF at 6 months, %	65 ± 7	68 ± 9	0.331
E/e′ at ablation	9.8 ± 3.5	9.9 ± 4.7	0.858
E/e′ at 3 months	7.9 ± 3.9	8.2 ± 5.8	0.974
E/e′ at 6 months	7.7 ± 3.4	8.4 ± 4.8	0.560
Reduction of LAD at 3 months, %	8.5 (4.4–13.2)	5.7 (− 1.2–12.5)	0.093
Reduction of LAD at 6 months, %	7.4 (3.8–13.4)	4.3 (0.0–10.2)	0.022

*LAD* left atrial dimension, *LVEF* left ventricular ejection fraction

### Outcome

**Fig. 4:**
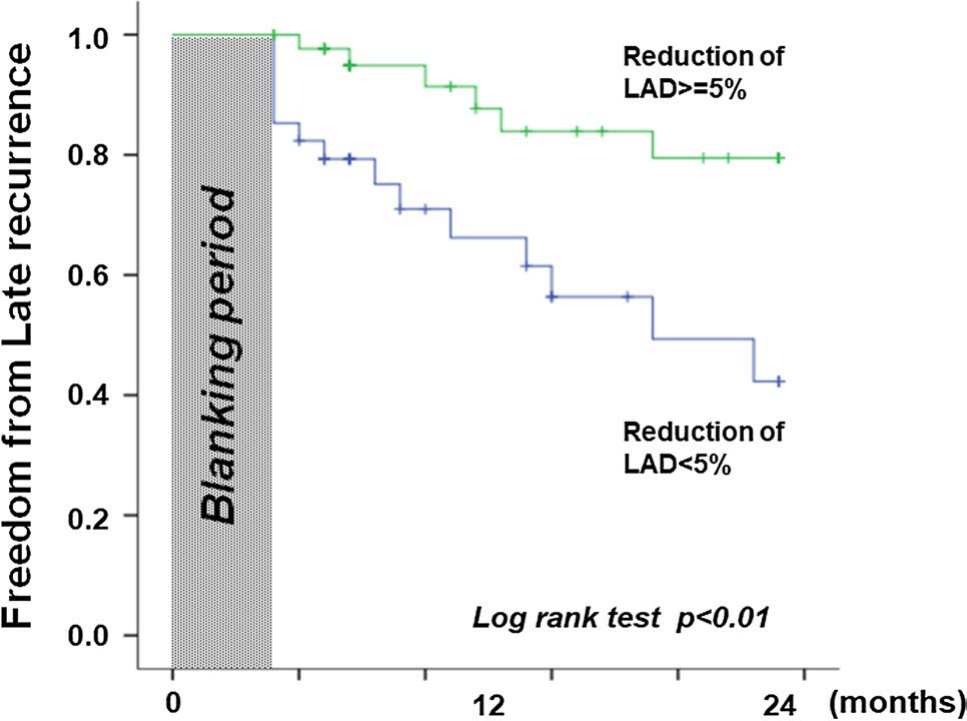
Kaplan–Meier analysis of long-term freedom from atrial fibrillation (AF) in patients with and without 5% reduction of left atrial dimension at 6 months after AF ablation. *LAD* left atrial dimension

Cox regression analysis revealed that a reduction of LA dimension ≥ 5% at 6 months was associated with freedom from late recurrence of AF (Table 4). At month 24 of the follow-up period excluding blanking periods, 80% of the patients with a LA dimension reduction ≥ 5% at 6 months were free from AF, compared with 42% of the patients without (Log-rank test, p < 0.01) (Fig. 4).

**Table 4:** Results of Cox regression analysis for freedom from AF recurrence

Covariates	*β*	Standard error	*P* value	Hazard ratio
Age	− 0.010	0.028	0.725	0.990
Male	− 0.531	0.670	0.404	0.588
Duration of AF	0.010	0.008	0.240	1.010
≥ 5% reduction of LAD	− 1.228	0.560	0.028	0.293
ERAF	0.642	0.525	0.221	1.900
Continuation of AADs	0.775	0.517	0.134	2.170

*AADs* antiarrhythmic drugs, *AF* atrial fibrillation, *ERAF* early recurrence of atrial fibrillation, *LAD* left atrial dimension

## Discussion

This study demonstrated that a reduction of more than 5% in LA dimension at 6 months after ablation was associated with freedom from late recurrence after catheter ablation of persistent AF.

It was previously reported that the prognostic factors of AF ablation include ERAF, AF duration, LA size and hypertension [13–16]. The durations of AF and ERAF significantly affected the outcomes after AF ablation in our study, as in previous reports.

Reverse morphological remodeling of the LA by ablation for isolated AF was observed in several studies [4, 13, 14]. Tops et al. reported that the LA antero-posterior diameter showed a significant reduction in their SR group at 3 months of follow-up [13]. Beukema et al. reported that the LA dimension in persistent AF subjects who remained in sinus rhythm decreased from 44.0 ± 5.8 to 40.0 ± 4.5 mm at 6 months after ablation, while in patients with recurrence of AF the LA dimension increased from 45 ± 6.5 to 49 ± 5.4 mm [14]. A reduced or eliminated AF burden could lead to a reduction of LA volume after catheter ablation [17, 18]. In our study, a reduction in LA volume at 6 months after ablation was seen in the patients who remained in sinus rhythm for the 2-year follow-up period.

Wu et al. reported that catheter ablation of AF improves structural remodeling of the PV ostia and LA. In that study, patients with persistent AF had a greater reduction in LA volume than those who had paroxysmal AF (11.8 ± 12.8 cm^3^ vs 4.0 ± 11.2 cm^3^; *P* = 0.04). Reverse remodeling of the PV ostial geometry may parallel the reduction of LA volume [18]. From an electrocardiographic point of view, Fujimoto et al. reported that P wave dispersion was useful for assessment of reverse remodeling after catheter ablation of AF [19].

We believe that the change in LA diameter may be used as a marker of AF recurrence, indicating continued use of antiarrhythmic drugs or the need for re-ablation. Based on our results, a smaller degree of reversibility can be expected in subjects with longer AF duration, due to progressive remodeling.

### Clinical implications

The identification of a strong predictor of AF recurrence (i.e., reduction of LA diameter) makes it possible to distinguish patients with different long-term success probabilities. This stratification may help to identify patients who would benefit from antiarrhythmic drugs or a repeat procedure to prevent recurrent AF.

### Limitations

This was a single-center study, and any conclusions must be regarded with appropriate circumspection. LA volume measured by CT or MRI in AF ablation was reported previously [20, 21]; although these modalities are more precise, we could easily speculate on the recurrence of AF based on whether LA remodeling after ablation was detected by two-dimensional echocardiography. The left atrial volume index (LAVI) has been measured since December 26, 2016 in our hospital and echocardiographic image by which the LAVI could be measured has not been recorded. Thus LAVI could not be analyzed in this study. Twenty-four patients (18%) underwent re-ablation for AF recurrence, which may introduce strong patient selection bias. Another major limitation is that this was a retrospective study; prospective confirmation will add further value.

## Conclusions

This study demonstrated that a higher rate of LA size reduction at 6 months after ablation of persistent AF was associated with a higher maintenance rate of sinus rhythm in the late phase. This is a new predictor for maintenance of sinus rhythm after ablation of persistent AF.
